# The effect of local variation in malaria transmission on the prevalence of sulfadoxine–pyrimethamine resistant haplotypes and selective sweep characteristics in Malawi

**DOI:** 10.1186/s12936-015-0860-7

**Published:** 2015-10-05

**Authors:** Elena Artimovich, Atupele Kapito-Tembo, Paul Pensulo, Osward Nyirenda, Sarah Brown, Sudhaunshu Joshi, Terrie E. Taylor, Don Mathanga, Ananias A. Escalante, Miriam K. Laufer, Shannon Takala-Harrison

**Affiliations:** Howard Hughes Medical Institute/Center for Vaccine Development, University of Maryland School of Medicine, 685 West Baltimore Street, HSF1-480, Baltimore, MD 21201 USA; Malaria Alert Center, Blantyre, Malawi; Blantyre Malaria Project, University of Malawi College of Medicine, Blantyre, Malawi; University of Maryland, Baltimore County, Baltimore, MD USA; Institute for Genomics and Evolutionary Medicine, Temple University, Philadelphia, PA USA

**Keywords:** Malaria, Sulfadoxine–pyrimethamine, Resistance, Selective sweeps, Dihydrofolate reductase (DHFR) and dihydropteroate synthase (DHPS)

## Abstract

**Background:**

Persistence of sulfadoxine–pyrimethamine (SP) resistance has been described in an urban setting in Malawi where malaria transmission is relatively low. Higher malaria transmission is associated with greater genetic diversity and more frequent genetic recombination, which could lead to a more rapid re-emergence of SP-sensitive parasites, as well as more rapid degradation of selective sweeps. In this study, the impact of local variation in malaria transmission on the prevalence of SP-resistant haplotypes and selective sweep characteristics was investigated at an urban site with low parasite prevalence and two rural sites with moderate and high parasite prevalence.

**Methods:**

Samples from three sites with different parasite prevalence were genotyped for resistance markers within *pfdhfr*-*ts* and *pfdhps* and at microsatellites flanking these genes. Expected heterozygosity (H_e_) was estimated to evaluate genetic diversity.

**Results:**

No difference in the prevalence of highly resistant DHFR 51I/59R/108N and DHPS 437G/540E was found between sites. Small differences in H_e_ flanking *pfdhfr*-*ts* and *pfdhps* were seen between rural-moderate and the other sites, as well as some shared haplotypes between the rural-high and urban-low sites.

**Conclusions:**

The results do not show an effect of local variation in malaria transmission, as inferred from parasite prevalence, on SP-resistant haplotype prevalence.

**Electronic supplementary material:**

The online version of this article (doi:10.1186/s12936-015-0860-7) contains supplementary material, which is available to authorized users.

## Background

The potential expansion of artemisinin-resistant *Plasmodium falciparum*, from Asia to Africa, has heightened interest in identifying factors affecting resistance allele dynamics, including the re-emergence of drug-sensitive malaria parasites. Chloroquine and sulfadoxine–pyrimethamine (SP) were both once used as safe and effective primary treatment for uncomplicated malaria. Successive waves of anti-malarial resistance from Southeast Asia prompted public health organizations to abandon chloroquine, and then SP, in favour of artemisinin-based combination therapy (ACT).

After the removal of chloroquine drug pressure in Malawi in 1993, chloroquine-sensitive parasites re-expanded in the population, eventually outcompeting the chloroquine-resistant strains [1]. The return of chloroquine sensitivity was shown, via the analysis of selective sweeps, to be the result of the re-emergence of genetically diverse sensitive parasites that had survived selective pressure [2]. This relatively rapid and nationwide re-emergence of genetically diverse drug-sensitive parasites in Malawi was unexpected, as drug resistance had persisted in South America and Southeast Asia many years after reduction in drug pressure [1, 3, 4].

In late 2007, Malawi replaced SP with an ACT in response to failing SP efficacy. The persistence of the highly resistant haplotypes of dihydrofolate reductase (DHFR) and dihydropteroate synthase (DHPS), DHFR 51I/59R/108N and DHPS 437G/540E, 5 years after the reduction of SP drug pressure, was recently demonstrated by the authors, with discussion of possible causes and implications of continued resistance [5]. Selective sweep analysis suggested little to no fitness cost of SP resistance in the urban setting of Ndirande where local transmission intensity is presumed relatively low.

The effect of reduction of SP use on SP-resistant parasites has been variable in other regions. While the persistence of SP-resistant parasites in Venezuela was shown years after the removal of drug pressure [6], a study of the frequency of Peruvian DHFR and DHPS resistance haplotypes showed a decline in DHFR 51I/108N/164L haplotypes within 5 years of SP removal as well as a decline in DHPS 437G/540E/581E [7]. Another recent study in Kenya observed differential change in genotype prevalence between rural-moderate and rural-high transmission settings 2 years after replacing SP with an ACT [8]. It is possible that these differences could be due to different background rates of malaria transmission.

Malaria parasites from areas of high transmission tend to be more diverse and undergo more recombination [6, 9, 10]. In the absence of selective pressure, high parasite diversity and recombination rates begin to increase the level of heterozygosity observed around resistance loci [6, 11]. Recombination between resistant and sensitive parasites from diverse lineages will begin to increase heterozygosity within the resistant parasite population, decreasing the width and depth of the selective sweep [6, 11]. Higher malaria transmission also results in a larger proportion of clinically immune hosts that can serve as a reservoir of drug-sensitive parasites that can re-expand after removal of drug pressure [12]. Areas with low malaria transmission tend to have lower levels of parasite diversity, leading to higher rates of inbreeding and more rapid rates of fixation of polymorphisms, possibly explaining why re-emergence of sensitive parasites was not seen in Venezuela. The rate of malaria transmission throughout Malawi is higher than that observed in South America and Southeast Asia, yet relatively higher and lower transmission settings can be found in rural and urban environments within Malawi. In this study the authors estimate the prevalence of DHFR and DHPS resistance haplotypes and the characteristics of the associated selective sweeps at three sites with low, moderate and high parasite prevalence within Malawi to explore whether local variation in transmission impacts the re-expansion of sensitive parasites.

## Methods

Samples were collected as part of a malaria surveillance study conducted in health facilities in three districts in Malawi: a rural site near a river with high parasite prevalence (Chikwawa), a rural more arid site with moderate parasite prevalence (Thyolo) and an urban site with low parasite prevalence (Ndirande). Samples were collected from individuals presenting to the hospital with uncomplicated malaria. All samples were collected with informed consent according to an institutional review board approved protocol. Samples consisted of blood spots, collected on filter papers, representing a patient’s initial infection on the day they were admitted to the study, prior to treatment. Parasite prevalence from a community-based, cross-sectional survey was used as a surrogate measure of transmission intensity; 8.4, 14.3 and 29.6 % of individuals surveyed in the urban-low, rural-moderate and rural-high settings, respectively, were qPCR-positive for *P. falciparum* parasites. Recent entomological inoculation rates (EIR) were not available for these sites, but the use of parasite prevalence as a surrogate for EIR and transmission intensity is supported in previous studies [13].

DNA was extracted from filter paper blood cards using a Qiagen BioRobot (Qiagen, Valencia, CA, USA) following the Investigator Bloodcard Protocol. Parasite genotypes at polymorphic sites within *pfdhfr*-*ts* and *pfdhps* genes were determined via pyrosequencing. Single nucleotide polymorphisms (SNPs) within codons 51, 59 and 108, of *pfdhfr*-*ts* and codons 437, 540 and 581 of *pfdhps* were genotyped for all samples using primers and amplification methods adapted from Zhou et al. [14]. Pyrosequencing was performed on a PyroMark Q96 MD system (Biotage, Charlotte, NC, USA). Allele frequency was adjusted based on a standard curve [15]. An allele with a relative frequency of 80 % or greater within a given infection was designated as the predominant allele. Haplotypes were constructed using only the predominant allele. Samples without a predominant allele at two or more codons were labelled ‘mixed-genotype’. Samples that were mixed at a single codon were treated as containing both possible haplotypes.

To show that reduced heterozygosity around drug resistance genes was the result of selection rather than demographic processes, expected heterozygosity was measured in six unlinked neutral loci located throughout the *P. falciparum* genome [16]. These unlinked microsatellites were amplified using previously published primers and amplification conditions [16]. When multiple peaks were identified within the same sample, peaks that were less than one-third the height of the tallest peak were ignored and the tallest peak was designated as the predominant allele [16, 17]. Only samples with a predominant allele were included in estimates of expected heterozygosity.

Eight polymorphic microsatellites flanking *pfdhfr*-*ts* were genotyped; four downstream (+50, +20, +1.48, +0.2 kb), and four upstream (−0.3, −1.2, −10, −30 kb) using previously described primers and protocols [11, 18]. Eight polymorphic microsatellites flanking *pfdhps* were genotyped; four downstream (+9.008, +1.407, +0.505, +0.034 kb), and four upstream (−0.132, −2.849, −7.489, −11.069 kb) of the gene, using primers described by Vinayak et al. [19]. Fragment size was visualized using an Applied BioSystems 3730XL high-throughput 96-capillary DNA sequencer. Analysis of electropherograms was performed using Genemapper software (version 4.0; ABI). A Perl script was used to assign the raw electropherogram scores to an integer allele size based on the expected repeat length and variation seen in the positive controls.

The prevalence of each haplotype was estimated as the number of each haplotype observed among the successfully genotyped samples for each resistance gene, divided by the total number of genotyped samples. Haplotype frequency, the proportion of parasites with a given haplotype, could not be calculated as the number of parasite clones per infection was not estimated. A resistant haplotype was defined as containing any number or combination of resistance alleles at the genotyped codons within either of the resistance genes of interest. The sensitive haplotype was defined as parasites with sensitive alleles at all codons within both genes. Samples with mixed-genotype at two or more loci within the same gene were excluded because haplotype phase could not be determined. Chi squared tests with Yate’s correction were used, where appropriate.

Expected heterozygosity (H_e_), a measure of genetic diversity at each microsatellite locus, was calculated using the standard equations for H_e_ and variance:

$$H_{e} = \left( {\frac{n}{n - 1}} \right)\left( {1 - \sum {p_{i}^{2} } } \right),\quad \frac{2(n - 1)}{{n^{3} }}\left\{ {2(n - 2)\left[ {\sum {p_{i}^{3} - \left( {\sum {p_{i}^{2} } } \right)} } \right]} \right\}$$

The analysis focused on sweep characteristics flanking DHFR 51I/59R/108N and DHPS 437G/540E due to limited prevalence of other haplotypes in all three settings. H_e_ (±1 standard deviation) was calculated for three groups: rural high, rural-moderate and urban-low sites, for both *pfdhfr*-*ts* and *pfdhps* genes. Samples without a predominant genotype or samples with missing data were excluded from expected heterozygosity calculations. Statistical significance was determined via permutation. Diversity ratios were calculated for H_urban-low_/H_rural-high_, H_urban-low_/H_rural-moderate_, and H_rural-moderate_/H_rural-high_. Calculations for H_e_, standard deviation and permutations were conducted in R [20].

## Results

**Fig. 1:**
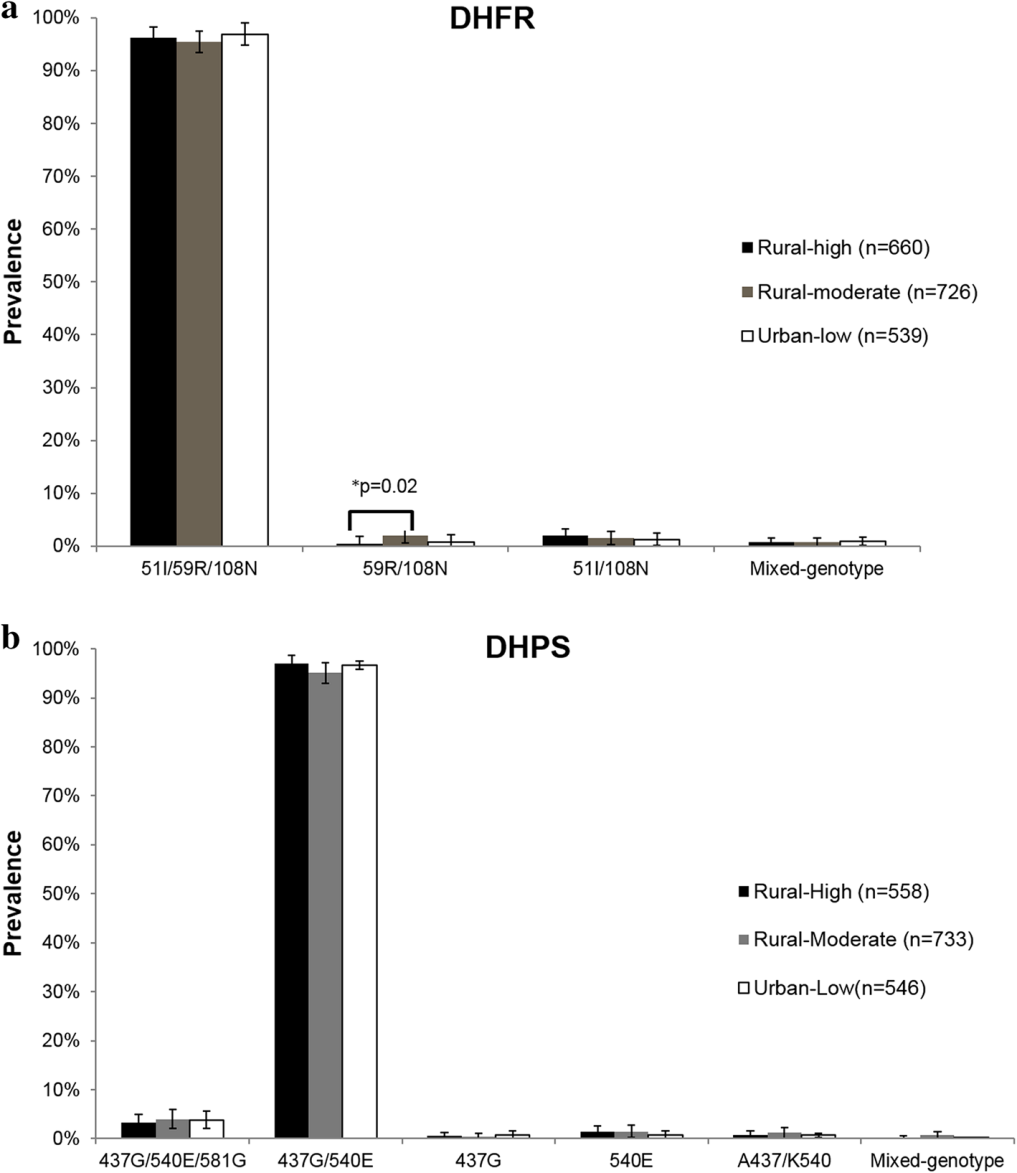
Sulfadoxine–pyrimethamine-resistant haplotype prevalence at three sites with different prevalence of malaria parasites. Haplotype prevalence at DHFR (**a**) and DHPS (**b**) at three sites within Malawi: a rural site with high parasite prevalence (Chikwawa), a rural site with moderate parasite prevalence (Thyolo), and an urban site with low parasite prevalence (Ndirande). Prevalence was calculated as the percentage of individuals with a given haplotype. *Error bars* represent 95 % confidence intervals

Of the available samples, complete *pfdhfr*-*ts* haplotypes were assembled for 549 samples from the urban-low site, 726 samples from the rural-moderate site, and 660 samples from the rural-high site (Fig. 1a). Complete *pfdhps* haplotypes were assembled for 546 samples from the urban-low site, 733 samples from the rural-moderate site and 558 samples from the rural-high site (Fig. 1b). No difference in the prevalence of the highly resistant DHFR 51I/59R/108N or DHPS 437G/540E was found between the three study sites. Haplotype prevalence of the DHFR 51I/59R/108N haplotype was >95 % for all study sites. The prevalence of the highly resistant DHPS 437G/540E was also >95 % for all three sites. A greater prevalence of DHFR 59R/108N was found in the rural-moderate site, relative to the rural-high site (0 vs 2 %, Yate’s corrected p = 0.020), and was borderline different from the urban-low site (1 vs 2 %, p = 0.067). A single, sensitive parasite infection was identified in the rural-moderate site.

**Fig. 2:**
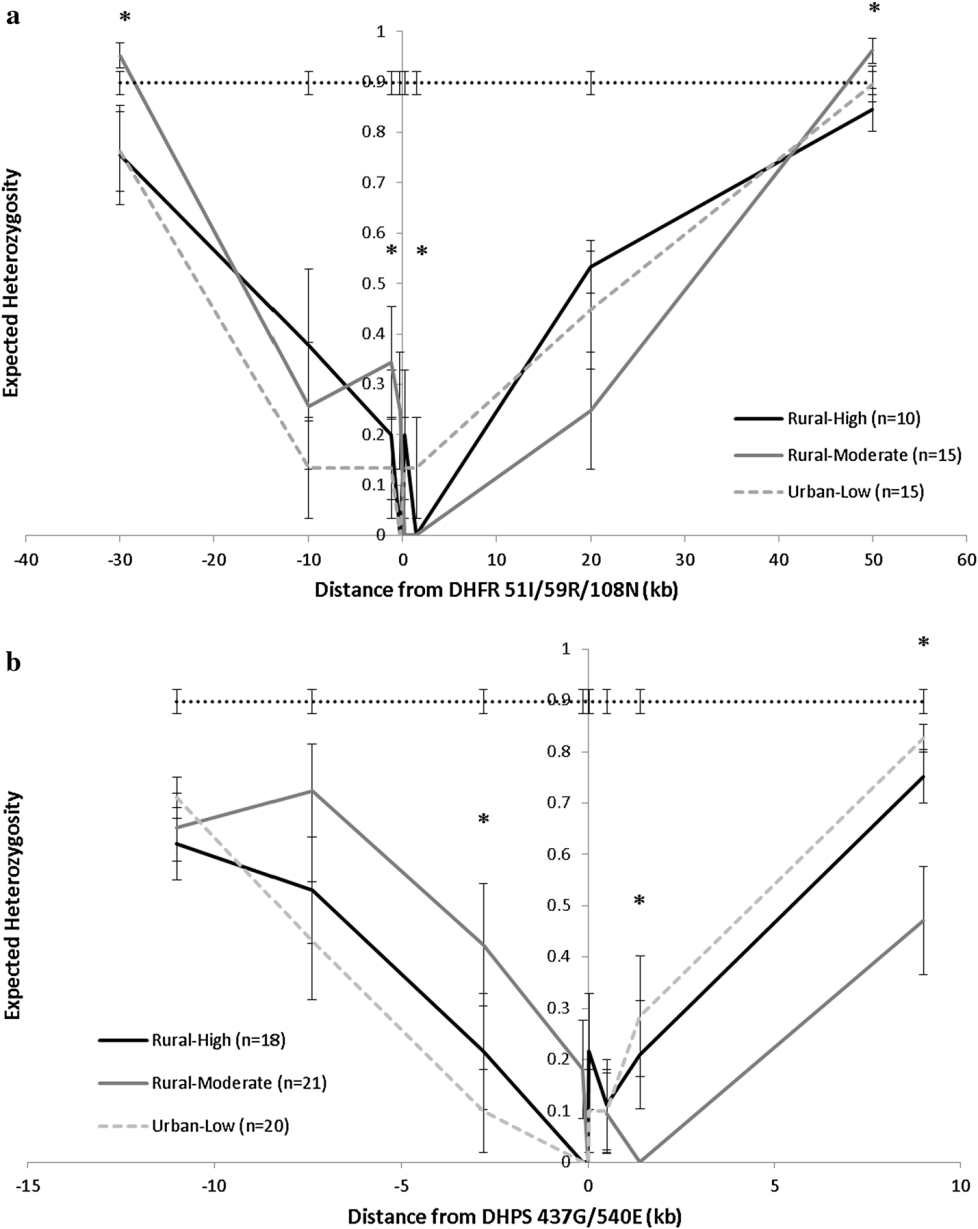
Sulfadoxine–pyrimethamine selective sweep characteristics at three sites with different prevalence of malaria parasites. Expected heterozygosity in microsatellite loci flanking DHFR 51I/59R/108N (**a**) and DHPS 437G/540E (**b**). Samples with missing data were excluded. *Error bars* represent ±1 standard deviation. *Dashed lines* represent genomic level, average H_e_, based on unlinked loci. Alpha <0.05 *asterisk* indicates significant difference between two study sites based on permutation

**Fig. 3:**
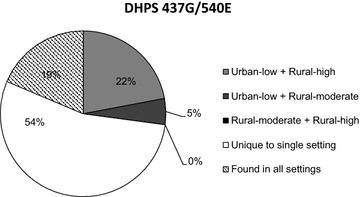
Proportion of unique versus common microsatellite haplotypes between study sites. Proportion of microsatellite haplotypes found between study sites

A sub-set of samples was subjected to microsatellite analyses to examine selective sweep characteristics between study sites. Of the sub-set, complete microsatellite haplotypes were generated for the rural-moderate site (n = 15, *pfdhfr*-*ts*), (n = 21, *pfdhps*), rural-high site (n = 10, *pfdhfr*-*ts*), (n = 18, *pfdhps*), and urban-low site (n = 15, *pfdhfr*-*ts*) and (n = 20, *pfdhps*). Average H_e_ at unlinked microsatellites was 0.898. The proportion of samples scored as polyclonal based on unlinked microsatellites did not differ significantly between study sites (p = 0.611). None of the sites had H_e_ values that were consistently different from each other (Fig. 2). Significant changes in H_e_, were seen between the rural-moderate site, and the rural-high site and urban-low site (p < 0.001) though no differences between the rural-high and urban-low sites were found. Analysis of flanking microsatellites indicated the presence of a core haplotype flanking *pfdhfr*-*ts* and *pfdhps,* likely of Southeast Asian origin, in all three sites (see Additional file 1). Lastly, microsatellite haplotypes flanking *pfdhps* found in the urban-low and rural-high sites were observed that were not present in the rural-moderate site, and microsatellite haplotypes found in the rural-moderate site not found in the other two locations (Fig. 3).

## Discussion

This study estimates the effect of local differences in malaria transmission, as inferred from parasite prevalence, on the prevalence of SP-resistant haplotypes and the characteristics of associated selective sweeps within Malawi. Despite marked differences in the parasite prevalence between the rural-high and urban low sites, no difference in haplotype prevalence or sweep characteristics was observed. Statistically significant differences between the prevalence of DHFR double mutants in the rural-moderate and rural-high sites are possibly a statistical artifact due to large sample size and are not considered clinically relevant.

This study also indicated similarity in sweep characteristics and some shared microsatellite haplotypes between the rural-high and urban-low sites, counter to what would be expected under the assumption that higher transmission would result in greater genetic diversity. These findings may indicate a shared parasite population between these locations. The divergence of some sweep characteristics and microsatellite haplotypes in the rural-moderate site compared to the other two sites, suggests that regional demographic events may affect sweep characteristics, without a significant effect on haplotype prevalence. Such events could have been the migration of SP-resistant haplotypes of diverse genetic background to the rural-moderate site (perhaps from neighbouring Mozambique).

The similarity in the proportion of samples scored as polyclonal between study sites may indicate similarity in the level of malaria transmission, not reflected in the parasite prevalence observed at each site. Recent EIRs were unavailable for these study sites; therefore, parasite prevalence was used as a surrogate for transmission intensity. If parasite prevalence was an accurate estimation of transmission intensity in this study, a larger proportion of polyclonal samples in the higher transmission sites would have been expected. This discrepancy could indicate that parasite prevalence is not a good surrogate for transmission intensity in this study. The estimation of transmission intensity used here was based on average parasite prevalence from a community-based, district-wide, cross-sectional survey, while the data-set used in this study was based on a facility-based survey. The cross-sectional data provide a single estimate of parasite prevalence at a given point in time, and may not reflect historical differences in transmission intensity between the sites. It is possible that individuals reporting to the district health centres in this study may have been from areas with more similar malaria transmission than the district-wide average parasite prevalence suggests. It is also possible that the almost three-fold difference in parasite prevalence between these sites is not sufficient to produce a measurable difference in the proportion of polyclonal infections, haplotype prevalence, or sweep characteristics.

The data also indicate a Southeast Asian origin of the Malawian *pfdhfr*-*ts* haplotype based on similarity in microsatellite repeat length between Malawian parasites and the V1S control strain. While, the Malawian *pfdhps* haplotype in this study differed at several markers from the V1S strain, core markers (those in close proximity to *pfdhps*) were similar to those published by Mita et al., and Alam et al. showing similar core haplotypes found in Malawi, Bangladesh, Thailand and Cambodia [21, 22]. The lack of published microsatellite data for reference strains makes direct comparison between publications difficult, but these data suggest a Southeast Asian origin of the *pfdhps* resistance allele in Malawi.

This research presents evidence that after nearly two decades of high SP resistance in Malawi, differences in regional malaria transmission, as inferred from parasite prevalence, do not impact the prevalence of SP-resistant parasite haplotypes. The SP-resistant haplotypes, DHFR 51I/59R/108N and DHPS 437G/540E, have maintained their high prevalence long after the removal of SP as the first-line treatment in multiple settings in Malawi. Investigators working in epidemiologically similar settings can reasonably assume similarity in SP-resistant haplotype prevalence and sweep characteristics between local sites with different parasite prevalence.

## Additional file

10.1186/s12936-015-0860-7 **DHFR and DHPS microsatellite haplotypes.** Description: Table of DHFR and DHPS microsatellite haplotypes. Reference strains V1S, 3D7 and HB3. “C”=Chikwawa, rural-high, “T”=Thyolo, rural-moderate, and “N”=Ndirande, urban-low. Mita 2011 (22) and Alam 2011 (21) refer to other publications which provide microsatellite haplotypes identified and shared between Southeast Asian and Malawian parasites at the pfdhps locus.

## Abbreviations

ACT: artemisinin-based combination therapy
*pfdhfr*-*ts*: dihydrofolate reductase-thymidylate synthase (gene)
*pfdhps*: dihydropteroate synthase (gene)
DHFR: dihydrofolate reductase
DHPS: dihydropteroate synthase
EIR: entomological inoculation rate
ICEMR: International Center for Excellence in Malaria Research
PCR: polymerase chain reaction
qPCR: quantitative polymerase chain reaction
SNP: single nucleotide polymorphism
SP: sulfadoxine–pyrimethamine

## References

[1] Laufer MK, Thesing PC, Eddington ND, Masonga R, Dzinjalamala FK, Takala SL (2006). Return of chloroquine antimalarial efficacy in Malawi. N Engl J Med.

[2] Laufer MK, Takala-Harrison S, Dzinjalamala FK, Stine OC, Taylor TE, Plowe CV (2010). Return of chloroquine-susceptible falciparum malaria in Malawi was a reexpansion of diverse susceptible parasites. J Infect Dis.

[3] Wellems TE, Plowe CV (2001). Chloroquine-resistant malaria. J Infect Dis.

[4] Dondorp AM, Nosten F, Yi P, Das D, Phyo AP, Tarning J (2009). Artemisinin resistance in *Plasmodium falciparum* malaria. N Engl J Med.

[5] Artimovich E, Schneider K, Taylor TE, Kublin JG, Dzinjalamala FK, Escalante AA (2015). Persistence of sulfadoxine–pyrimethamine resistance despite reduction of drug pressure in Malawi. J Infect Dis.

[6] McCollum AM, Mueller K, Villegas L, Udhayakumar V, Escalante AA (2007). Common origin and fixation of *Plasmodium falciparum* dhfr and dhps mutations associated with sulfadoxine–pyrimethamine resistance in a low-transmission area in South America. Antimicrob Agents Chemother.

[7] Zhou Z, Griffing SM, de Oliveira AM, McCollum AM, Quezada WM, Arrospide N (2008). Decline in sulfadoxine–pyrimethamine-resistant alleles after change in drug policy in the Amazon region of Peru. Antimicrob Agents Chemother.

[8] Vardo-Zalik AM, Zhou G, Zhong D, Afrane YA, Githeko AK, Yan G (2013). Alterations in *Plasmodium falciparum* genetic structure two years after increased malaria control efforts in western Kenya. Am J Trop Med Hyg.

[9] Paul RE, Hackford I, Brockman A, Muller-Graf C, Price R, Luxemburger C (1998). Transmission intensity and *Plasmodium falciparum* diversity on the northwestern border of Thailand. Am J Trop Med Hyg.

[10] Lumb V, Das MK, Singh N, Dev V, Khan W, Sharma YD (2011). Multiple origins of *Plasmodium falciparum* dihydropteroate synthetase mutant alleles associated with sulfadoxine resistance in India. Antimicrob Agents Chemother.

[11] Nash D, Nair S, Mayxay M, Newton PN, Guthmann JP, Nosten F (2005). Selection strength and hitchhiking around two anti-malarial resistance genes. Proc Biol Sci.

[12] Snow RW, Omumbo JA, Lowe B, Molyneux CS, Obiero JO, Palmer A (1997). Relation between severe malaria morbidity in children and level of *Plasmodium falciparum* transmission in Africa. Lancet.

[13] Manjurano A, Okell L, Lukindo T, Reyburn H, Olomi R, Roper C (2011). Association of sub-microscopic malaria parasite carriage with transmission intensity in north-eastern Tanzania. Malar J.

[14] Zhou Z, Poe AC, Limor J, Grady KK, Goldman I, McCollum AM (2006). Pyrosequencing, a high-throughput method for detecting single nucleotide polymorphisms in the dihydrofolate reductase and dihydropteroate synthetase genes of *Plasmodium falciparum*. J Clin Microbiol.

[15] Lumb V, Das MK, Singh N, Dev V, Wajihullah, Sharma YD (2009). Characteristics of genetic hitchhiking around dihydrofolate reductase gene associated with pyrimethamine resistance in *Plasmodium falciparum* isolates from India. Antimicrob Agents Chemother.

[16] Nair S, Williams JT, Brockman A, Paiphun L, Mayxay M, Newton PN (2003). A selective sweep driven by pyrimethamine treatment in southeast asian malaria parasites. Mol Biol Evol.

[17] Lim P, Chy S, Ariey F, Incardona S, Chim P, Sem R (2003). pfcrt polymorphism and chloroquine resistance in *Plasmodium falciparum* strains isolated in Cambodia. Antimicrob Agents Chemother.

[18] Roper C, Pearce R, Bredenkamp B, Gumede J, Drakeley C, Mosha F (2003). Antifolate antimalarial resistance in southeast Africa: a population-based analysis. Lancet.

[19] Vinayak S, Alam MT, Mixson-Hayden T, McCollum AM, Sem R, Shah NK (2010). Origin and evolution of sulfadoxine resistant *Plasmodium falciparum*. PLoS Pathog.

[20] Development CTR. R: a language and environment for statistical computing. R Foundation for Statistical Computing. 2008.

[21] Alam MT, Vinayak S, Congpuong K, Wongsrichanalai C, Satimai W, Slutsker L (2011). Tracking origins and spread of sulfadoxine-resistant *Plasmodium falciparum dhps* alleles in Thailand. Antimicrob Agents Chemother.

[22] Mita T, Venkatesan M, Ohashi J, Culleton R, Takahashi N, Tsukahara T (2011). Limited geographical origin and global spread of sulfadoxine-resistant *dhps* alleles in *Plasmodium falciparum* populations. J Infect Dis.

