# Supplementary material for: The effect of local variation in malaria transmission on the prevalence of sulfadoxine–pyrimethamine resistant haplotypes and selective sweep characteristics in Malawi
Source: Malar J. 2015 Oct 5;14:387. doi: 10.1186/s12936-015-0860-7 (PMC4595317; doi:10.1186/s12936-015-0860-7)
Supplement: Supplementary file 1 — 10.1186/s12936-015-0860-7 DHFR and DHPS microsatellite haplotypes. Description: Table of DHFR and DHPS microsatellite haplotypes. Reference strains V1S, 3D7 and HB3. “C”=Chikwawa, rural-high, “T”=Thyolo, rural-moderate, and “N”=Ndirande, urban-low. Mita 2011 (22) and Alam 2011 (21) refer to other publications which provide microsatellite haplotypes identified and shared between Southeast Asian and Malawian parasites at the pfdhps locus. [file 12936_2015_860_MOESM1_ESM.docx]

## Additional Figure 1: DHFR and DHPS microsatellite haplotypes. Reference strains V1S, 3D7 and HB3. “C”=Chikwawa, rural-high, “T”=Thyolo, rural-moderate, and “N”=Ndirande, urban-low. Mita 2011 (22) and Alam 2011 (21) refer to other publications which provide microsatellite haplotypes identified and shared between Southeast Asian and Malawian parasites at the *pfdhps* locus.
